# Standard of care in advanced HIV disease: review of HIV treatment guidelines in six sub-Saharan African countries

**DOI:** 10.1186/s12981-023-00581-5

**Published:** 2023-11-23

**Authors:** Thomas C. Scheier, Nabila Youssouf, Mosepele Mosepele, Cecilia Kanyama, Olukemi Adekanmbi, Sulaiman Lakoh, Conrad K. Muzoora, Graeme Meintjes, Dominik Mertz, John W. Eikelboom, Sean Wasserman

**Affiliations:** 1 Population Health Research Institute, McMaster University and Hamilton Health Sciences, Hamilton, ON Canada; 2 Botswana-Harvard AIDS Institute Partnership, Gaborone, Botswana; 3 University of North Carolina Project-Malawi, Lilongwe, Malawi; 4 Department of Medicine, College of Medicine, University of Ibadan, Ibadan, Nigeria; 5 College of Medicine and Allied Health Sciences, University of Sierra Leone, Freetown, Sierra Leone; 6 Department of Internal Medicine Faculty of Medicine Mbarara, University of Science and Technology Mbarara, Mbarara, Uganda; 7 Centre for Infectious Diseases Research in Africa, Institute of Infectious Disease and Molecular Medicine, University of Cape Town, Cape Town, South Africa; 8 Department of Medicine, University of Cape Town, Cape Town, South Africa; 9 Division of Infectious Diseases, Department of Medicine, McMaster University, Hamilton, ON Canada; 10 Department of Health Research Methodology, Evidence, and Impact, Faculty of Health Sciences, McMaster University, Hamilton, ON Canada; 11 Institute for Infection and Immunity, St George’s, University of London, London, UK

**Keywords:** Advanced HIV disease, Standard of care, Sub-Saharan Africa, REVIVE trial, WHO

## Abstract

**Background:**

The World Health Organization (WHO) recommends an evidence-based package of care to reduce mortality and morbidity among people with advanced HIV disease (AHD). Adoption of these recommendations by national guidelines in sub-Saharan Africa is poorly documented. We aimed to review national guidelines for AHD management across six selected countries in sub-Saharan Africa for benchmarking against the 2021 WHO recommendations.

**Methods:**

We reviewed national guidelines from six countries participating in an ongoing randomized controlled trial recruiting people with AHD. We extracted information addressing 18 items of AHD diagnosis and management across the following domains: [1] Definition of AHD, [2] Screening, [3] Prophylaxis, [4] Supportive care, and [5] HIV treatment. Data from national guideline documents were compared to the 2021 WHO consolidated guidelines on HIV and an agreement score was produced to evaluate extent of guideline adoption.

**Results:**

The distribution of categories of agreement varied for the national documents. Four of the six countries addressed all 18 items (Malawi, Nigeria, Sierra Leone, Uganda). Overall agreement with the WHO 2021 guidelines ranged from 9 to 15.5 out of 18 possible points: Malawi 15.5 points, Nigeria, and Sierra Leone 14.5 points, South Africa 13.5 points, Uganda 13.0 points and Botswana with 9.0 points. Most inconsistencies were reported for the delay of antiretroviral therapy (ART) in presence of opportunistic diseases. None of the six national guidelines aligned with WHO recommendations around ART timing in patients with tuberculosis. Agreement correlated with the year of publication of the national guideline.

**Conclusion:**

National guidelines addressing the care of advanced HIV disease in sub-Saharan Africa are available. Besides optimal timing for start of ART in presence of tuberculosis, most national recommendations are in line with the 2021 WHO standards.

## Background

Despite enormous advances in antiretroviral therapy (ART) and prevention of opportunistic infections, about 650 000 people died from human immunodeficiency virus (HIV)-related causes in 2021. The World Health Organization (WHO) African Region is the most affected, contributing to 25.6 million people living with HIV (PWH) and 420 000 HIV-related deaths in 2021 [1, 2]. The mortality burden largely comprises adults with advanced HIV disease (AHD), defined as CD4 + count < 200 cells/mm^3^ or a WHO stage 3 or 4 event [3], who experience 10 to 20% mortality [4].

The number of patients with AHD in sub-Saharan Africa is persistently high. In some areas more than half of people with a new diagnosis of HIV present with advanced disease [5–8] and an increasing number of people presenting with AHD are ART experienced [9]. Mortality in this group is mainly driven by opportunistic infections including tuberculosis, cryptococcal meningitis, and severe bacterial infection, all of which are potentially preventable and curable [3, 10].

To reduce the burden of preventable morbidity and mortality, and related health care costs in people with AHD, the WHO in 2017 published the first international guideline providing evidence-based diagnostic, prophylaxis, and treatment recommendations for people with AHD to be delivered as a package of care [3]. To translate WHO guidance into health outcomes, specific recommendations need to be adopted into national practice guidelines and implemented in ART programs. It is unclear to what extent evidence-based WHO recommendations on AHD have been adopted in national guidelines.

The objective of this study is to review and document national guidelines for AHD management across selected countries in sub-Saharan with a high burden of advanced HIV for benchmarking against WHO recommendations. Findings may help to identify gaps in care and areas for health system investment.

## Methods

### Search strategy

Investigators representing six sub-Saharan African countries (Botswana [11], Malawi [12], Nigeria [13], South Africa [14], Sierra Leone [15], and Uganda [16]) participating in an ongoing trial of azithromycin prophylaxis for AHD (“REVIVE”, NCT05580666) were asked to provide published national guideline documents that included recommendations on management of AHD. We also searched the WHO website for the most recent guideline documents that related to management of AHD and included two publications for comparison [3, 17].

### Data extraction

To compare the different documents (two WHO documents, six national documents) for management of AHD, we defined 18 items grouped in six categories: [1] Definition of AHD (1 item), [2] Screening (6 items), [3] Prophylaxis (6 items), [4] Supportive care (1 item) and [5] HIV treatment (4 items) (Tables 1, 2, 3). Available information was extracted from each guideline document and entered onto a spreadsheet for analysis. The national investigator of each country reviewed and confirmed the accuracy of data extraction from national guidelines and one author (SW) independently reviewed and confirmed the accuracy of data extraction from the WHO guidelines.

**Table 1:** Extracted data country specific guidelines

	**WHO 2021**	Botswana	Malawi	Nigeria	Sierra Leone	South Africa	Uganda
Guideline information							
Country specific guideline	**N/A**	Yes	Yes	Yes	Yes	Yes	Yes
Year of publication	**2021**	2016	2022	2020	2020	2023	2018
Specific section for AHD	**Yes**	No	No	Yes	No	No	Yes
Definition of AHD							
Definition	** < 200 CD 4 + cells/mm**^**3**^** or WHO clinical stage 3 or 4**	≤ 100 CD 4 + cells/mL^3^ or WHO clinical stage 3 & 4	< 200 CD 4 + cells/mm^3^ or WHO clinical stage 3 or 4 or additional criteria*	< 200 CD 4 + cells/mm^3^ or WHO clinical stage 3 or 4	≤ 200 CD 4 + cells/mm^3^ or WHO clinical stage 3 or 4	Not addressed	< 200 CD 4 + cells/mm^3^ or WHO clinical stage 3 or 4
Screening							
CD4 testing at baseline	**Yes**	Yes	Yes	Yes	Yes	Yes	Yes
Cryptococcal antigen	**Yes**	Yes	Yes	Yes	Yes	Yes	Yes
- Population group	- **Recommended: < 100 CD 4 + cells/mm**^**3**^ - **Considered: < 200 CD 4 + cells/mm**^**3**^	< 100 CD 4 + cells/mm^3^	AHD	< 200 CD 4 + cells/mm^3^	- < 100 CD 4 + cells/mm^3^ All PLHIV on ART suspected or - confirmed to have treatment failure	< 100 CD 4 + cells/mm^3^	- Positive symptom screen or - Danger sign - or ≤ 100 CD 4 + cells/mm^3^
Routine TB screening	**Yes**	Yes	Yes	Yes	Yes	Yes	Yes
- Urine LAM	**Yes**	Not addressed	Yes	Yes	Yes	Yes	Yes
○ Population group	- **Inpatients ≤ 200 CD 4 + cells/mm**^**3**^ - **Outpatients ≤ 100 CD 4 + cells/mm**^**3**^ - **Any CD 4 + count** **with symptoms or if seriously ill**^**#**^	Not addressed	AHD	< 200 CD 4 + cells/mm^3^	< 100 CD 4 + cells/mm^3^	- CD4 count < 200 within the last 6 months, or - Advanced HIV disease, or - Current serious illness	- TB symptoms and ≤ 100 CD 4 + cells/mm3 - Danger signs at any CD 4 + count
Prophylaxis							
Cotrimoxazole	**Yes**	Yes	Yes	Yes	Yes	Yes	Yes
- Population group	- < **350 CD 4 + cells/mm**^**3**^** or clinical stage 3 or 4** - **Any CD4 count in settings with high prevalence of malaria or severe bacterial infection**	< 200 CD 4 + cells/mm^3^	All HIV infected adults	Any CD 4 + cell or WHO stage, due to high prevalence of malaria and severe bacterial infections	All HIV infected adults	CD 4 + count ≤ 200 cells/mm^3^, WHO Stage 2, 3 and 4	- All PLHIV newly initiating on ART - Patients suspected to have treatment failure
- Discontinuation	- **Clinically stable on ART**^**+**^**, with evidence of immune recovery and/or viral suppression**^**$,&**^ - **Malaria and /or severe bacterial infections are highly prevalent: co-trimoxazole prophylaxis should be continued regardless of CD4 cell count or WHO clinical stage**	> 200 CD 4 + cells/mm^3^ for three months	For life	May be discontinued in adults who are clinically stable on ART with evidence of immune recovery and virological suppression	For life	CD 4 + count > 200 cells/mm^3^	- Patient should be older than 15 years of age - Patient should not be pregnant - Patient should have been on ART for at least one year - Patient’s last VL should be suppressed - Patient should not have a treatment WHO stage 3 or 4 event at the time of stopping CPT or other symptoms of Advanced Disease
Pre-emptive therapy for cryptococcal antigenaemia	**Yes**	Yes	Yes	Yes	Yes	Yes	Yes
TB preventive therapy	**Yes**	Not addressed	Yes	Yes	Yes	Yes	Yes
- Population group	**Any**	Not addressed	HIV infected adults	Any CD4 cell count	All HIV patients	Any CD4 count	Negative symptom screen and any CD 4 + count
Supportive care							
Specified adherence information	**Yes**	Yes	Yes	Yes	Yes	Yes	Yes
HIV treatment							
Recommended 1st line ART	**DTG/3TC/TDF OR DTG/FTC/TDF**	DTG/FTC/TDF	TDF/3TC/DTG	DTG/3TC/TDF OR DTG/FTC/TDF	DTG/3TC/TDF	DTG/3TC/TDF	DTG/3TC/TDF
Delayed/Deferred ART start after start of TB/cryptococcal treatment							
- TB at non-neurological site	**within 2 weeks**	ART naïve - < 100 CD 4 + cells/mm3 as soon as possible, no later than 8 weeks - > 100 CD 4 + cells/mm3 within 8 weeks	Within 2 weeks	- if < 50 CD 4 + cells/mm^3^ 2 weeks - if > 50 CD 4 + cells/mm^3^ 4 weeks	2–8 weeks	DS-TB: - < 50 CD 4 + cells/mm^3^ within 2 weeks - ≥ 50 CD 4 + cells/mm^3^ after 8 weeks DR-TB: - 2 weeks	- if < 50 CD 4 + cells/mm^3^ within 2 weeks - if > 50 CD 4 + cells/mm^3^ 2 weeks
- TB meningitis	**4– 8 weeks**	- < 50 CD 4 + cells/mm^3^ ART within first two weeks - < 100 CD 4 + cells/mm^3^ as soon as possible, no later than 8 weeks - > 100 CD 4 + cells/mm^3^ within 8 weeks	5 weeks	4 weeks	2–8 weeks	4–8 weeks	- if < 50 CD 4 + cells/mm^3^ within 2 weeks if > 50 CD 4 + cells/mm^3^ 2 weeks
- Cryptococcal meningitis	**4**–**6 weeks**	4–6 weeks	5 weeks	4–6 weeks	4–6 weeks	4–6 weeks	4–6 weeks

*3TC* Lamivudine, *AHD* advanced HIV Disease, *ART* anti retroviral therapy, *AZT* Zidovudine, *DTG* Dolutegravir, *FTC* Emtricitabine, *LAM* lipoarabinomannan, *N/A* not applicable, *TDF* Tenofovir dixoproxil fumerate, *DS-TB* drug-sensitive tuberculosis, *DS-TB* drug-resistant tuberculosis

Bold: reference (WHO 2021)

^*^Every ART experienced patient with viral load 1000 + (on ART for > 1 year); seriously ill: all PLHIV admitted as in-patient; HIV infected patients with any of the following danger signs: adults: ≥ 30 breaths/min; heart rate ≥ 120 beats/min; unable to walk unaided; ≥ 39 °C

^#^Seriously ill: a seriously ill adult is defined as having any of the following danger signs: respiratory rate ≥ 30 breaths per minute; heart rate ≥ 120 beats per minute; or unable to walk unaided. Other clinical conditions, such as body temperature ≥ 39 °C, can also be considered based on local epidemiology and clinical judgement

^+^Clinically stable adults are defined as individuals receiving ART for at least one year without any new WHO clinical stage 2, 3, or 4 events

^$^CD4 cell count > 350 cells/mm^3^, with suppression of viral loads, is considered immune recovery (some countries may adopt a threshold of CD4 cell count > 500 cells/mm3)

^&^WHO recognizes that in settings with low prevalence of malaria and severe bacterial infection in which co-trimoxazole is used primarily as prophylaxis for some AIDS-associated opportunistic infections (Pneumocystis jirovecii pneumonia and toxoplasmosis), guidelines exist for adults living with HIV discontinuing co-trimoxazole when there is evidence of suppressed viral loads and immune recovery at CD4 cell count > 200 cells/mm^3^ and they have been receiving ART for at least one year

**Table 2:** Extracted data WHO documents

	WHO 2017	**WHO 2021**
Guideline information		
Country specific guideline	N/A	**N/A**
Year of publication	2017	**2021**
Specific section for AHD	Yes	**Yes**
Definition of AHD		
Definition	< 200 CD 4 + cells/mm^3^ or WHO clinical stage 3 or 4	** < 200 CD 4 + cells/mm**^**3**^** or WHO clinical stage 3 or 4**
Screening		
CD4 testing at baseline	Yes	**Yes**
Cryptococcal antigen	Yes	**Yes**
- Population group	- CD 4 + cell count < 100 cells/mm^3^	- **Recommended: CD4 < 100 cells/mm**^**3**^ - **Considered: CD4 < 200 cells/mm**^**3**^
Routine TB screening	Yes	**Yes**
- Urine LAM	Yes	**Yes**
○ Population group	- Symptoms suggesting TB and who have CD 4 + count ≤ 100 cells/mm^3^ - At any CD4 count if seriously ill^#^	- **Inpatients ≤ 200 CD 4 + cells/mm**^**3**^ - **Outpatients ≤ 100 CD 4 + cells/mm**^**3**^ - **Any CD 4 + count** **with symptoms or if seriously ill**^**#**^
Prophylaxis		
Cotrimoxazole	Yes	**Yes**
- Population group	- ≤ 350 CD 4 + cells/mm3 or clinical stage 3 or 4 - Any CD 4 + count in settings with high prevalence of malaria or severe bacterial infections	- **CD 4 + < 350 cells/mm**^**3**^** or clinical stage 3 or 4** - **Any CD 4 + count in settings with high prevalence of malaria or severe bacterial infection**
- Discontinuation	- Clinically stable on ART, with evidence of immune recovery and viral suppression - Malaria and /or severe bacterial infections are highly prevalent: co-trimoxazole prophylaxis should be continued regardless of CD 4 + cell count or WHO clinical stage	- **Clinically stable on ART**^**+**^**, with evidence of immune recovery and/or viral suppression**^**&$**^ - **Malaria and /or severe bacterial infections are highly prevalent: co-trimoxazole prophylaxis should be continued regardless of CD 4 + cell count or WHO clinical stage**
Pre-emptive therapy for cryptococcal antigenemia	Yes	**Yes**
TB preventive therapy	Yes	**Yes**
- Population group	Any	**Any**
Supportive care		
Specified adherence information	Yes	**Yes**
HIV treatment		
Recommended 1st line ART	Not addressed	**DTG/3TC/TDF OR DTG/FTC/TDF**
Delayed ART start		
- TB at non-neurological site	- Within first 8 weeks - If CD 4 + cell counts < 50 cells/mm^3^ start within 2 weeks	**Within 2 weeks**
- TB meningitis	2 months	**4–8 weeks**
- Cryptococcal meningitis	4**–**6 weeks	**4–6 weeks**

Bold: reference (WHO 2021)

WHO 2017: guidelines for managing advanced HIV disease and rapid initiation of antiretroviral therapy July 2017

WHO 2021: consolidated guidelines on HIV prevention, testing, treatment, service delivery and monitoring: recommendations for a public health approach July 2021

WHO 2023: providing care to people with advanced HIV disease who are seriously ill policy brief

^#^Seriously ill: a seriously ill adult is defined as having any of the following danger signs: respiratory rate ≥ 30 breaths per minute; heart rate ≥ 120 beats per minute; or unable to walk unaided. Other clinical conditions, such as body temperature ≥ 39 °C, can also be considered based on local epidemiology and clinical judgement

^+^Clinically stable adults are defined as individuals receiving ART for at least one year without any new WHO clinical stage 2, 3, or 4 events

^&^CD4 cell count > 350 cells/mm^3^, with suppression of viral loads, is considered immune recovery (some countries may adopt a threshold of CD4 cell count > 500 cells/mm3)

^$^WHO recognizes that in settings with low prevalence of malaria and severe bacterial infection in which co-trimoxazole is used primarily as prophylaxis for some AIDS-associated opportunistic infections (Pneumocystis jirovecii pneumonia and toxoplasmosis), guidelines exist for adults living with HIV discontinuing co-trimoxazole when there is evidence of suppressed viral loads and immune recovery at CD4 cell count > 200 cells/mm^3^ and they have been receiving ART for at least 1 year

**Table 3:**
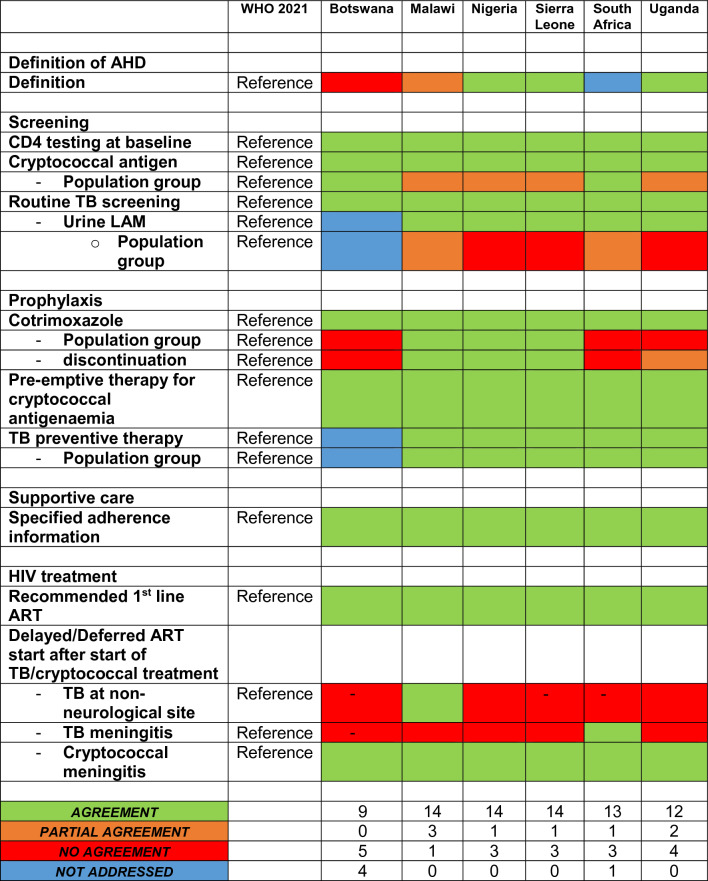
Agreement of country specific guidelines to WHO 2021

Green: agreement; red: no agreement; orange: patrial agreement; blue: not addressed

### Evaluation of guideline agreement

All documents were compared to a WHO reference guideline. We categorized items in each guideline as follows:

*Not addressed*. No information for the respective item was found within the national guidelines.

*Agreement*. The extracted data for an item in the country guideline matched the *WHO 2021* guideline recommendations.

*Partial agreement.* The data of the national guideline included at least the same, but non-identical, criteria (e.g., *WHO 2021*: CD4 +  < 100 cells/mm^3^, National guideline: CD4 +  < 200 cells/mm^3^).

*No agreement*. National recommendation not in line with the *WHO 2021* guidelines.

We used a scoring system to quantitatively rate the agreement. Agreement was assigned 1-point, Partial agreement 0.5-points, and No agreement and Not addressed 0-points.

### Analysis

The categories of overall agreement were displayed as bar graphs. Points on the scoring system were aggregated for each document to obtain an overall score.

## Results

### Reference guideline documents

Data from following WHO documents was extracted:

- 2017: “Guidelines for managing advanced HIV disease and rapid initiation of antiretroviral therapy” published in 2017 (hereinafter called *WHO 2017*). This guideline provides specific recommendations about management of people presenting with AHD and timing of initiation of ART for all PWH [3].
- 2021: “Consolidated guidelines on HIV prevention, testing, treatment, service delivery and monitoring: recommendations for a public health approach” published in 2021 (hereinafter called *WHO 2021*). This document includes existing and new clinical and programmatic recommendations and brings together all relevant WHO guidance on HIV produced since 2016. Information about HIV prevention, testing, treatment, service delivery and monitoring is provided across different ages, populations and settings [17].

*WHO 2021* was selected as a reference to define the gold standard for guideline comparison. This document was chosen because it is a consolidated guideline including all relevant WHO guidance on HIV produced since 2016 and therefore also incorporated and superseded *WHO 2017*. “Providing care to people with advanced HIV disease who are seriously ill” [18] was published in 2023 as a policy brief and was not included in this manuscript.

*WHO 2021* provides recommendations for all defined items (Tables 1 and 2). Agreement with the national guidelines is shown in Table 3.

### National guideline documents

Data was extracted from six national guidelines (Table 4). All countries established country specific guidelines, which were published between 2016 and 2023. Only the guidelines of Nigeria and Uganda include a specific section for AHD. The extracted data from national guidelines is presented in Table 1.

**Table 4:** Index of national guidlines

Country	Name	References
Botswana	Handbook of the Botswana 2016 integrated HIV clinical care guidelines	(1)
Malawi	Clinical Management of HIV in children and adults	(2)
Nigeria	National guidelines for HIV prevention, treatment and care	(3)
Sierra Leone	Consolidated guidelines on HIV prevention, diagnosis, treatment and care in Sierra Leone	(4)
South Africa	2023 ART clinical guidelines for the management of HIV in Adults, pregnancy and breastfeeding, adolescents, children, infants and neonates, version 4	(5)
Uganda	Consolidated guidelines for the prevention and treatment of HIV in Uganda	(6)

1. Ministry of Health Republic of Botswana. Handbook of the Botswana 2016 integrated HIV clinical care guidelines. 2016

2. Ministry of Health and Population Malawi. Clinical management of HIV in children and adults. 2022

3. National AIDS and STIs Control Programme Federal Ministru of Health Nigeria. National guidelines for HIV prevention, treatment and care. 2020

4. Ministry of Health and Sanitation Sierra Leone. Consolidated guidelines on HIV prevention, diagnosis, treatment and care in Sierra Leone. 2020

5. Republic of South Africa National Department of Health. 2023 ART Clinical Guidelines for the management of HIV in adults, pregnancy, adolescents, children, infants and neonates. 2023, Version 4

6. Ministry of Health Republic of Uganda. Consolidated guidelines for the prevention and treatment of HIV in Uganda. 2018; Second Edition

### Definition AHD

*WHO 2021* defines advanced HIV as CD4 + count < 200 cells/mm^3^ or WHO clinical stage 3 or 4. Nigeria, Sierra Leone, and Uganda use the same definitions. Botswana uses a CD4 + cut-off of less than 100 cells/mL and Malawi includes additional criteria for the diagnosis, including virological failure, hospitalization status and clinical danger signs. South African guidelines do not provide a definition of AHD.

### Screening for opportunistic infections

agreement was found for at least four of the six items for all countries. All national guidelines recommend testing for CD4 + at baseline, if available. However, guidelines differ in their recommendations for screening for cryptococcal antigen and tuberculosis (Tables 1 and 3). Both screening procedures are recommended in every document, but the targeted population groups vary slightly.

### Cryptococcal screening

According to *WHO 2021*, screening for cryptococcal disease is recommended in PWH and a CD4 +  < 100 cells/mm^3^ and should be considered in PWH with CD4 < 200 cells/mm^3^. Recommendations of Botswana and South Africa are in line with *WHO 2021*. Malawi, Nigeria, Sierra Leone, and Uganda screen a wider population group than recommended. Malawi includes all patients with AHD, Nigeria uses a threshold of CD4 + 200 cells/mm^3^, and Sierra Leone also screens PWH on ART with suspected or confirmed treatment failure. Uganda assesses clinical information, either positive symptom screening or danger signs, or a CD4 + count < 100 cells/mm^3^.

### Tuberculosis screening

*WHO 2021* recommends screening for tuberculosis with urine lipoarabinomannan (LAM) for inpatients (CD4 +  < 200 cells/mm^3^) or outpatients (CD4 +  < 100 cells/mm^3^), any CD4 count with symptoms, or if seriously ill. Routine tuberculosis symptom screening is recommended in all countries. The use of urine LAM is not addressed in the guideline documents from Botswana. None of the remaining countries uses an approach stratified by CD4 + cell count for in- and outpatient LAM screening. In contrast to *WHO 2021,* Nigeria and Sierra Leone do not include clinical criteria as an indication for urine LAM testing, both using a CD4 + threshold of 100 cells/mm^3^ only. Malawi includes all patients with AHD, and South Africa recommends urine LAM screening for patients with CD4 count < 200 cells/mm^3^ within the last 6 months, AHD or current serious illness.

### Prophylaxis of opportunistic infections

Malawi, Nigeria, Sierra Leone (all agreement), South Africa (4 agreement, 2 no agreement), and Uganda (4 agreement, 1 partial agreement, 1 no agreement) addressed all six items. Botswana addressed only 4 items, of which two were described as no agreement.

### Cotrimoxazole prophylaxis

In *WHO 2021,* cotrimoxazole prophylaxis is recommended for PWH with a CD4 + count < 350 cells/mm^3^, clinical stage 3 or 4, or to any PWH in settings with high prevalence of malaria or severe bacterial infection. All national documents recommend use of cotrimoxazole, but the targeted population group varies. Sierra Leone, Nigeria, and Malawi include all HIV infected adults for cotrimoxazole prophylaxis, which is in line with *WHO 2021* because malaria or severe bacterial infection are highly prevalent in those countries. Botswana and South Africa use a lower CD4 + cut off (CD4 +  < 200 cells/mm^3^). Uganda recommends cotrimoxazole for all people newly initiating ART and for people with ART treatment failure (Table 1).

Malawi and Sierra Leone recommend lifelong prophylaxis with cotrimoxazole. In Nigeria, prophylaxis can be discontinued once clinically stable on ART. Both approaches are in line with *WHO 2021*. In national recommendations from Uganda five criteria need to be met to discontinue prophylaxis: age, pregnancy status, duration of ART, viral load, and current clinical status (Table 1). In contrast, Botswana and South Africa recommend discontinuing prophylaxis once CD4 + cell count reaches 200 cells/mm^3^.

### Tuberculosis preventive therapy

All national guidelines, except Botswana (not addressed), recommend preventive therapy for tuberculosis for all adults living with HIV.

### Supportive care interventions

Intensified treatment adherence support for people with AHD is recommended in all documents. Interventions include adherence counseling in Botswana or home visits—if feasible – in Nigeria.

### Antiretroviral therapy (ART)

All national documents addressed items in this domain. All national guidelines include dolutegravir (DTG), emtricitabine (FTC) or lamivudine (3TC), and tenofovir disoproxil fumarate (TDF) as the recommended first line regimen. Guidelines vary in recommendations to defer ART start in patients requiring treatment for tuberculosis.

### Start ART in PWH and tuberculosis

In contrast to *WHO 2021*, which makes recommendations without taking CD4 + levels into account (tuberculosis at non-neurological site, start ART within 2 weeks; tuberculosis meningitis, start at 4–8 weeks), Malawi, Nigeria, and Uganda require a CD4 + count to define when to start ART. South Africa further differentiates according to drug-sensitive or drug-resistant tuberculosis.

### Start ART in PWH and cryptococcal meningitis

All national documents agree with *WHO 2021* for timing of ART in cryptococcal meningitis, recommending a delayed start of ART for 4–6 weeks after diagnosis.

### Overall agreement

**Fig. 1:**
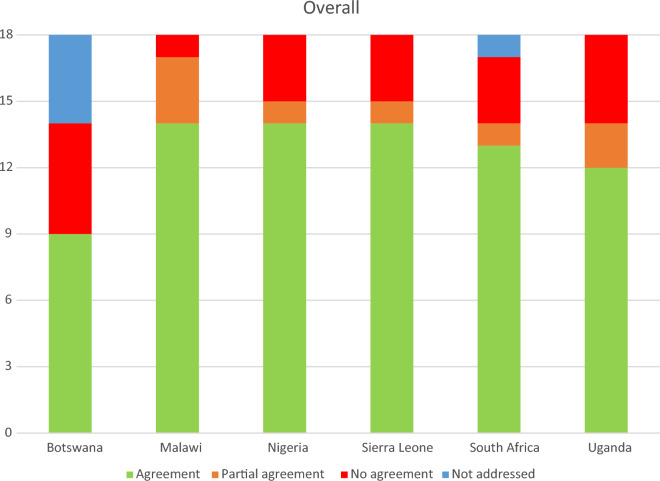
Categories of agreement for national guidelines

**Fig. 2:**
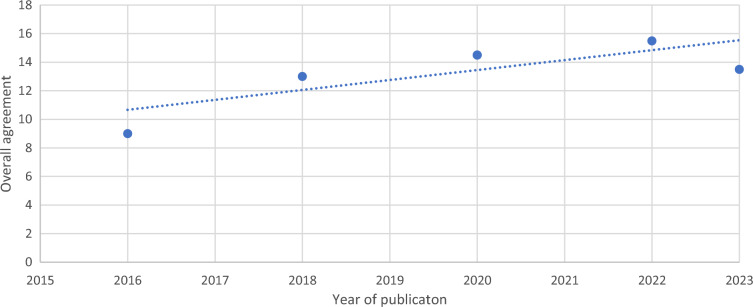
Overall agreement according to year of publication

Botswana obtained 9 points (9 Agreement, 5 No agreement, 4 Not addressed), Malawi 15.5 points (14 Agreement, 3 Partial agreement, 1 No agreement), Nigeria and Sierra Leone 14.5 points (14 Agreement, 1 Partial agreement, 3 No agreement), South Africa 13.5 points (13 Agreement, 1 Partial agreement, 3 No agreement, 1 Not addressed) and Uganda 13 points (12 Agreement, 2 Partial agreement, 4 No agreement) (Table 2, Fig. 1*)*. Alignment with *WHO 2021* recommendations varies over time, but shows an increasing trend (Fig. 2).

## Discussion

This review evaluates national guideline documents from six sub-Saharan African countries to describe the standard of care for AHD. Overall, national guidelines addressed between 14 and 18 items and at least 60% of the addressed items were in agreement with the WHO reference document across all six national documents.

Various enhanced clinical care approaches, including prophylaxis, screening procedures or intensified adherence counselling, have been shown to reduce mortality in AHD [19, 20]. We defined a set of 18 items to reflect these broad areas. All items were addressed in the national guidelines from Malawi, Nigeria, Sierra Leone, and Uganda. Gaps were documented in the guidelines from Botswana and South Africa.

The number of items not addressed at a national level (range 0–4) could be explained by local circumstances and policies or presence of other national documents dealing with defined topics of our items, such as national tuberculosis guidelines, that were not included in our review.

AHD is defined by CD4 + count and clinical stage. Only half of the country-specific guidelines were in line with the definition provided by *WHO 2021*. CD4 + testing at baseline is important to identify patients at highest risk for morbidity and mortality, especially since nearly half of people presenting with CD4 + counts < 100 cells/mm^3^ have WHO clinical stage 1 or 2 [19]. However, there has been a decrease of pre-ART CD4 + testing in the treat-all era in low/lower-middle-income countries and the determination of CD4 count varies widely across Africa [21, 22]. Malawi is the only country to provide additional clinical criteria to define advanced HIV disease, a tacit and pragmatic acknowledgement that CD4 testing might not be available in many clinics in that country.

The agreement of national guidelines compared to *WHO 2021* varies. We established different categories of agreement to quantify and summarize differences; importantly, these categories are not a measure of clinical importance or guideline quality. The highest agreement was shown for Malawi, followed by Nigeria, and Sierra Leone. Most no agreement scores were in the ART domain, with all national documents showing differences in comparison to *WHO 2021*. The choice of first line ART is aligned in all documents, reflecting wide availability of highly effective and well tolerated drugs. In contrast, the optimal timing for ART initiation with concomitant tuberculosis does not align with WHO recommendations and varies across countries. This discrepancy may reflect a reluctance of countries to implement rapid (same day) ART initiation for PHW undergoing tuberculosis screening or within 2 weeks for those starting tuberculosis treatment given the increased risk of immune reconstitution inflammatory syndrome and the absence of mortality benefit for patients with CD4 + count > 50 cells/mm^3^ in randomized controlled trials [23].

The reviewed national guidelines were published between 2016 and 2023 and WHO recommendations published in 2017 and 2021. The changes seen in the WHO documents, such as indication for the use of urine LAM or timing of ART with tuberculosis, reflect the ongoing scientific advances in this area. Overall agreement between national documents and *WHO 2021* increased over time, reflecting the importance of providing regular updates to national guidelines in a rapidly changing field.

The strength of this survey is the close collaboration with leading clinicians and researchers situated in the evaluated countries. Furthermore, we included WHO documents, issued over a period of 6 years, to highlight changes in international guidance documents.

A limitation of this work is that only one guideline for each country was assessed, and relevant recommendations from other topic-specific guidelines may have been missed. Furthermore, we restricted our evaluation of national guidelines to the six countries participating in the vanguard phase of the REVIVE trial, limiting the generalizability of findings to other countries. The included countries however account for roughly 350 million people, representing nearly one third of the total population of sub-Saharan Africa [24]. Our study did not collect information around guideline implementation. It is estimated that only 28% of national policy documents of countries in the WHO African region adhere to the WHO HIV testing strategy [25] and that most of the 25 sub-Saharan countries do not appear to have widely implemented specific interventions regarding the care of AHD [26]. A study in Senegal showed that there are missed opportunities to prevent HIV-associated morbidity and mortality in AHD due to various barriers including diagnostic evaluation [27]. Implementation research to measure uptake and understand implementation barriers is needed.

## Conclusion

In conclusion, we found that national guidelines of six countries in sub-Saharan Africa address most recommendations relevant in the care of patients living with AHD. However, level of agreement with the 2021 WHO recommendations varied across countries. The largest discrepancies were in the recommendations for timing of ART in relation to tuberculosis. Encouragingly, agreement appears to be increasing over time.

## Data Availability

All data generated or analysed during this study are included in this published article [and its supplementary information files].

## Abbreviations

3TC: Lamivudine
AHD: Advanced HIV disease
ART: Antiretroviral therapy
DTG: Dolutegravir
FTC: Emtricitabine
HIV: Human immunodeficiency virus
LAM: Lipoarabinomannan
PWH: People living with HIV
TDF: Tenofovir disoproxil fumarate
WHO: World Health Organization

## References

[1] World Health Organization. HIV, Estimated number of people (all ages) living with HIV. https://www.who.int/data/gho/data/indicators/indicator-details/GHO/estimated-number-of-people--living-with-hiv. Accessed 20 July 2023.

[2] World Health Organization. HIV, Number of people dying from HIV-related causes. https://www.who.int/data/gho/data/indicators/indicator-details/GHO/number-of-deaths-due-to-hiv-aids. Accessed 20 July 2023.

[3] Guidelines for Managing Advanced HIV Disease and Rapid Initiation of Antiretroviral Therapy. WHO Guidelines Approved by the Guidelines Review Committee. Geneva; 2017 29341560

[4] Manosuthi W, Charoenpong L, Santiwarangkana C (2021). A retrospective study of survival and risk factors for mortality among people living with HIV who received antiretroviral treatment in a resource-limited setting. AIDS Res Ther.

[5] Leeme TB, Mine M, Lechiile K, Mulenga F, Mosepele M, Mphoyakgosi T (2021). Utility of CD4 count measurement in the era of universal antiretroviral therapy: an analysis of routine laboratory data in Botswana. HIV Med.

[6] Carmona S, Bor J, Nattey C, Maughan-Brown B, Maskew M, Fox MP (2018). Persistent high burden of advanced HIV disease among patients seeking care in South Africa's National HIV Program: data from a nationwide laboratory cohort. Clin Infect Dis.

[7] Lamp K, McGovern S, Fong Y, Atem CD, Nfetam JBE, Nzuobontane D (2020). Proportions of CD4 test results indicating advanced HIV disease remain consistently high at primary health care facilities across four high HIV burden countries. PLoS ONE.

[8] Stoger L, Katende A, Mapesi H, Kalinjuma AV, van Essen L, Klimkait T (2022). Persistent high burden and mortality associated with advanced HIV disease in rural tanzania despite uptake of World Health Organization "Test and Treat" guidelines. Open Forum Infect Dis.

[9] Osler M, Hilderbrand K, Goemaere E, Ford N, Smith M, Meintjes G (2018). The continuing burden of advanced HIV disease over 10 years of increasing antiretroviral therapy coverage in South Africa. Clin Infect Dis.

[10] Boyd AT, Oboho I, Paulin H, Ali H, Godfrey C, Date A (2020). Addressing advanced HIV disease and mortality in global HIV programming. AIDS Res Ther.

[11] Ministry of Health Republic of Botswana. Handbook of the Botswana 2016 integrated HIV clinical care guidelines. 2016.

[12] Ministry of Health and Population Malawi. Clinical management of HIV in children and adults. 2022.

[13] National AIDS and STIs Control Programme Federal Ministru of Health Nigeria. National Guidelines for HIV Prevention, Treatment and Care. 2020.

[14] Republic of South Africa National Department of Health. 2023 ART Clinical Guidelines for the Management of HIV in Adults, Pregnancy and Breastfeeding, Adolescents, Children, Infants and Neonates. June 2023 Version 4: Republic of South Africa National Department of Health; 2023.

[15] Ministry of Health and Sanitation Sierra Leone. Consolidated Guidelines on HIV Prevention, Diagnosis, Treatment and Care in Sierra Leone. 2020.

[16] Ministry of Health Republic of Uganda. Consolidated guidelines for the prevention and treatment of HIV in Uganda. 2018 Second Edition.

[17] Consolidated Guidelines on HIV Prevention (2021). Testing, treatment, service delivery and monitoring: recommendations for a Public Health Approach.

[18] World Health Organization (2023). Providing care to people with advanced HIV disease who are seriously ill: policy brief.

[19] Hakim J, Musiime V, Szubert AJ, Mallewa J, Siika A, Agutu C (2017). Enhanced prophylaxis plus antiretroviral therapy for advanced HIV infection in Africa. N Engl J Med.

[20] Mfinanga S, Chanda D, Kivuyo SL, Guinness L, Bottomley C, Simms V (2015). Cryptococcal meningitis screening and community-based early adherence support in people with advanced HIV infection starting antiretroviral therapy in Tanzania and Zambia: an open-label, randomised controlled trial. Lancet.

[21] Brazier E, Tymejczyk O, Zaniewski E, Egger M, Wools-Kaloustian K, Yiannoutsos CT (2021). Effects of national adoption of treat-all guidelines on pre-antiretroviral therapy (ART) CD4 testing and viral load monitoring after art initiation: a regression discontinuity analysis. Clin Infect Dis.

[22] Lakoh S, Kamudumuli PS, Penney ROS, Haumba SM, Jarvis JN, Hassan AJ (2023). Diagnostic capacity for invasive fungal infections in advanced HIV disease in Africa: a continent-wide survey. Lancet Infect Dis.

[23] Uthman OA, Okwundu C, Gbenga K, Volmink J, Dowdy D, Zumla A (2015). Optimal timing of antiretroviral therapy initiation for HIV-infected adults with newly diagnosed pulmonary tuberculosis: a systematic review and meta-analysis. Ann Intern Med.

[24] World Bank. Population, total: Worldbank; https://data.worldbank.org/indicator/SP.POP.TOTL?name_desc=false. Accessed 20 July 2023.

[25] Fonner VA, Sands A, Figueroa C, Baggaley R, Quinn C, Jamil MS (2020). Country adherence to WHO recommendations to improve the quality of HIV diagnosis: a global policy review. BMJ Glob Health.

[26] Izco S, Garcia-Basteiro AL, Denning DW, Boulware DR, Penn-Nicholson A, Letang E (2023). Management of advanced HIV disease in Africa. Lancet HIV.

[27] Benzekri NA, Sambou JF, Ndong S, Tamba IT, Faye D, Diallo MB (2019). Prevalence, predictors, and management of advanced HIV disease among individuals initiating ART in Senegal, West Africa. BMC Infect Dis.

